# Improved RRT*-Connect Manipulator Path Planning in a Multi-Obstacle Narrow Environment

**DOI:** 10.3390/s25082364

**Published:** 2025-04-08

**Authors:** Xueyi He, Yimin Zhou, Haonan Liu, Wanfeng Shang

**Affiliations:** 1 School of Mechanical and Control Engineering, Guilin University of Technology, Guilin 541006, China; 2120221180@glut.edu.cn (X.H.); 2120221198@glut.edu.cn (H.L.); 2 Shenzhen Institutes of Advanced Technology, Chinese Academy of Sciences, Shenzhen 518055, China; 3 National Engineering Laboratory for Big Data System Computing Technology, Shenzhen University, Shenzhen 518000, China; wf.shang@siat.ac.cn

**Keywords:** RRT*-Connect, heuristic sampling strategy, adaptive step size, node rejection strategy, path planning, robotic arm

## Abstract

This paper proposes an improved RRT*-Connect algorithm (IRRT*-Connect) for robotic arm path planning in narrow environments with multiple obstacles. A heuristic sampling strategy is adopted with the integration of the ellipsoidal subset sampling and goal-biased sampling strategies, which can continuously compress the sampling space to enhance the sampling efficiency. During the node expansion process, an adaptive step-size method is introduced to dynamically adjust the step size based on the obstacle information, while a node rejection strategy is used to accelerate the search process so as to generate a near-optimal collision-free path. A pruning optimization strategy is also proposed to eliminate the redundant nodes from the path. Furthermore, a cubic non-uniform B-spline interpolation algorithm is applied to smooth the generated path. Finally, simulation experiments of the IRRT*-Connect algorithm are conducted in Python and ROS, and physical experiments are performed on a UR5 robotic arm. By comparing with the existing algorithms, it is demonstrated that the proposed method can achieve shorter planning times and lower path costs of the manipulator operation.

## 1. Introduction

With the continuous advancement of production automation, robotic arms have been widely applied in various fields such as medical surgery, industrial manufacturing and agricultural harvesting, significantly improving production efficiency and quality [1,2]. On the production lines, robotic arms are often used for tasks such as module assembly, welding or grinding. To meet the requirements of these tasks, it is typically necessary to plan a collision-free continuous and optimal path for the robotic arm from the starting point to the target point under a given set of constraints [3].

The path planning of robotic arms in complex and narrow environments with multiple obstacles poses challenges, and path-planning algorithms can generally be categorized into three types: graph-based search, deep learning-based, and sampling-based algorithms [4]. In the graph-based search algorithms, such as A* [5] and D* algorithms [6], the search space is represented as a graph structure consisting of nodes and edges (paths connecting the nodes), aiming to find the optimal path from the initial point to the destination. While these algorithms are simple and easy to implement, they incur high computational costs and memory consumption when applied to high-dimensional path-planning scenarios [7]. Deep learning-based algorithms, however, can leverage the powerful nonlinear modeling capabilities of the deep neural networks to directly or indirectly learn the path-planning strategies from the environmental data. For instance, Meng et al. [8] have proposed a path-planning method which integrates deep learning with a sampling-based planner (NR-RRT), where risk profile maps and neural network-based samplers are employed to rapidly identify risk-constrained paths. Similarly, Huang et al. [9] have introduced a learning-based path-planning method, i.e., Neural-Informed RRT* (NIRRT*), where the point-cloud-based state representation under the acceptable ellipsoidal constraints with the PointNet++ network can directly generate multiple guiding states from the input point cloud of the free states and accelerate convergence to the optimal path by guiding the sampling process with neural networks. Although deep learning-based path-planning algorithms can improve planning efficiency, they require large amounts of data for training and inference, resulting in longer computational times and higher memory usage. In contrast, sampling-based algorithms, due to their stochastic nature, have lower computational costs and probabilistic completeness. Consequently, these algorithms have been widely adopted in robotic manipulator motion planning [10,11].

Sampling-based path-planning algorithms mainly include Probabilistic Roadmap (PRM) [12] and Rapidly Exploring Random Tree (RRT) [13]. The RRT is a single-query sampling-based path-planning method which incrementally explores the space by generating random nodes and extending a tree structure until a collision-free path from the initial point to the target point is found. Compared to the PRM, the RRT is more efficient and has lower time costs. However, the randomness in the RRT sampling and expansion would lead to slow convergence, and difficulty in navigating narrow passages and nonoptimal paths.

To address these issues, various improved RRT-based algorithms have been proposed in recent years. Kuffner and LaValle [14] proposed the RRT-Connect algorithm, which can simultaneously generate two trees at the initial and goal points. By employing a greedy strategy, the two trees grow towards each other, enabling faster pathfinding. However, the bidirectional search still exhibits a certain degree of randomness and cannot guarantee an optimal path obtainment. Karaman and Frazzoli [15] introduced RRT*, an extension of RRT, which incorporates the parental node reselection and rewiring mechanisms, achieving asymptotic optimality at the cost of increased computation time. Gammell et al. [16] proposed an Informed-RRT* algorithm with constrained sampling space to an ellipsoid after finding an initial path to improve the planning efficiency. However, it requires substantial time for narrow passage searches and is dependent on the quality of the initial path. Li et al. [17] proposed an Adaptive Step-size RRT* (AS-RRT*) algorithm, which employs an accumulator-based sampling point selection strategy to enhance the efficiency of the path planning and the pruning optimization methods to quickly identify collision-free paths, thereby reducing the computational costs. Zhang et al. introduced the HP-APF-RRT* algorithm to enhance the sampling efficiency and path search capability through heuristic probability sampling and artificial potential field (APF) methods [18].

The integration of RRT* with an APF method can enhance the planning efficiency in narrow passages, but it may be trapped in local optima [19]. Then a heuristic probability-biased goal (PBG-RRT) approach is proposed to achieve faster convergence with local minima avoidance [20]. Another Heuristic Dynamic Rapidly exploring Random Tree Connect (HDRRT-Connect) algorithm has been proposed to dynamically adjust the step size based on the environmental information so as to mitigate the local optimal path issues [21]. Dai et al. proposed a potential field extension strategy with a bidirectional tree structure and direct connection strategies to enhance the node expansion efficiency [22]. Jiang et al. employed a hybrid constraint sampling method to bring nodes closer to the narrow passages between the obstacles. During the expansion process, the adaptive gravitational fields, dynamic step sizes and constrained new node strategy are used to optimize the RRT-Connect algorithm, where these enhancements could effectively reduce excessive exploration and expansion in collision-prone areas [23]. Cheng et al. [24] employed an adaptive step-size strategy and fixed sampling functions to establish four trees, conducting searches from different starting points, thereby addressing the issue of the slow expansion speed in the RRT algorithm. Recent efforts include accelerating the path search with goal bias and adaptive step-size strategies [25] and the RRT* optimization with a manipulability-aware path-planning strategy to minimize the path costs [26]. The Adaptive Expansion Bidirectional RRT* (AEB-RRT*) [27] has been developed to dynamically adjust the sampling probabilities to speed up the convergence. Further, the unexplored area sampling probability is used for faster space exploration in narrow environments [28]. With the biased growth of bidirectional RRT-Connect trees and the Informed-RRT algorithm, the dynamic bias points can be applied to guide the tree growth for faster path generation [29].

Although numerous improvements have been made to the RRT-related path-planning algorithms, there remain shortcomings in terms of sampling space constraints and longer planning times. The lack of goal bias in the node expansion would limit the algorithm flexibility. Here, this paper proposes a path-planning algorithm based on the improved RRT*-Connect, considering the sampling space constraints, node expansion and path pruning in complex and multi-obstacle environments. The main contributions of the paper are summarized as follows:

(1) A heuristic sampling strategy which integrates the ellipsoid subset sampling and goal-biased sampling is proposed to improve the sampling efficiency in path planning. Initially, goal-biased sampling is utilized to identify an initial path. Then the sampling nodes are concentrated within the ellipsoidal region of the current optimal solution to narrow down the search space gradually and reduce exploration in invalid regions.

(2) An adaptive step-size expansion strategy is introduced to dynamically adjust the step size based on the known environment and the node information so as to reach the target region during the expansion, with reduced invalid exploration but predefined expansion into collision-prone areas. Moreover, a node rejection strategy is proposed to remove the invalid nodes by calculating the estimated cost of the new nodes.

(3) A path-pruning optimization approach is developed to remove the redundant nodes and smooth the path via the cubic non-uniform B-spline interpolation.

(4) We conduct experimental comparisons of the proposed algorithm with other algorithms in both 2D and 3D map environments to validate its superiority. Furthermore, the effectiveness of the algorithm is verified in the ROS simulation environment and practical robotic arm experiments.

The remainder of the manuscript is organized as follows. An exposition of the collision detection model and the underlying principles of the RRT*-Connect algorithm are introduced in Section 2. The improved variant of the RRT*-Connect path-planning algorithm is explained in Section 3. Simulation experiments and experimental validation with physical prototypes are shown in Section 4. Finally, the conclusion is provided in Section 5.

## 2. Background Formation

### 2.1. The Collision Detection Model

The Flexible Collision Library (FCL) [30] can be used for collision detection between rigid, deformable or articulated objects and point clouds or octrees, without requiring precise modeling during the collision detection process. However, the input models of the FCL must be sufficiently accurate. In the context of robotic arm collision avoidance, more time is often spent on modeling the robotic arm and acquiring the scenery point cloud data. Moreover, due to environmental noise and varied capture conditions, the obtained point clouds require extensive preprocessing. In practical engineering applications, obstacles usually have irregular shapes, and it is difficult to develop precise mathematical models.

**Figure 1:**
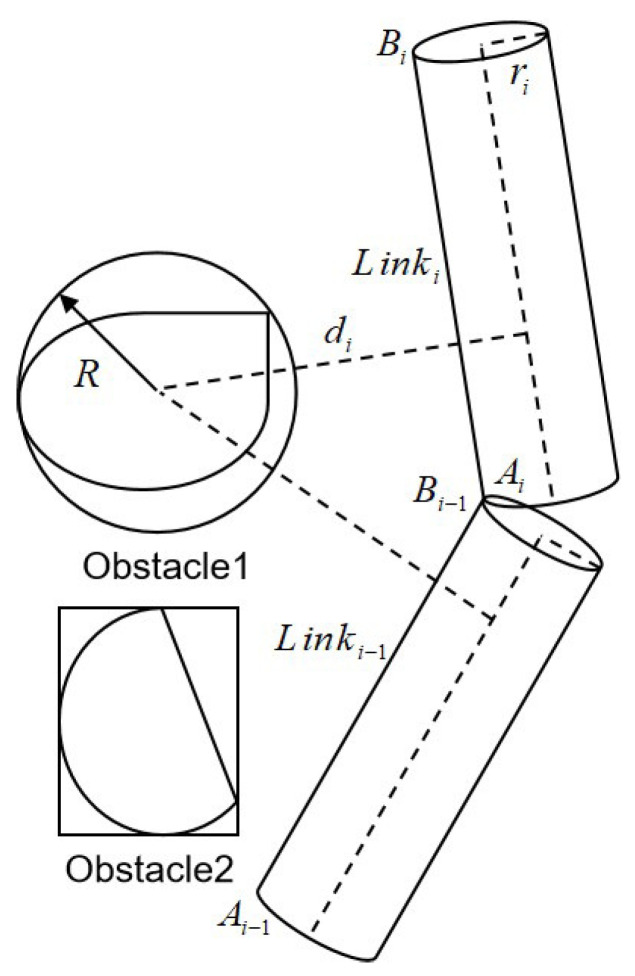
The robotic arm collision detection model.

Considering the algorithm development process and computational complexity for real-time collision detection, the direct modeling of the manipulator and obstacles can be simplified based on the concepts of bounding boxes and spatial overlays [31]. Here, this study adopts a spatial geometric envelope method to simplify the modeling of obstacles and the manipulator. The manipulator links are represented by cylinders, while irregular obstacles are approximated with spheres and rectangular prisms. The collision detection problem of the manipulator is then transformed into the detection of collisions among the cylinders, spheres and rectangular prisms. As demonstrated in Figure 1, the center of the sphere is indicated by the coordinates (x,y,z), where the cylinder represents the simplified model of the robotic arm with the two ends of $Link_{i}$ and a radius $r_{i}$. For the collision detection of the sphere boundary volume, the sphere represents a simplified model of obstacles with a radius *R*, and the distance from the center of the sphere to the center axis of the cylinder is $d_{i}$. When $d_{i}>r_{i}+R$, no collision occurs between the robotic arm and the obstacle; otherwise, a collision is detected. For the axis-aligned bounding box $A_{i}B_{i}$ collision detection, if the line segment is outside the obstacle $A_{i}B_{i}$, no collision occurs; otherwise, a collision is detected.

### 2.2. The RRT*-Connect Algorithm

Kuffner and LaValle proposed the RRT-Connect path-planning algorithm [14], which can accelerate the path convergence speed compared to the traditional RRT algorithm. However, this method lacks asymptotic optimality, and the RRT*-Connect path-planning algorithm is further proposed with RRT* and RRT-Connect in combination to optimize the path during the expansion [32].

**Figure 2:**
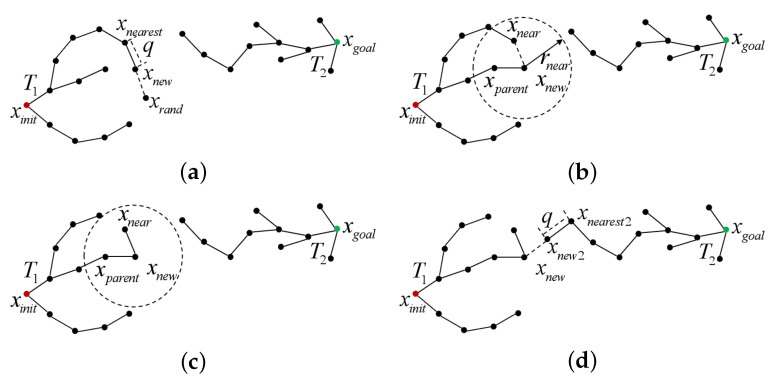
The workflow of the RRT*-Connect algorithm: (**a**) New Node Generation; (**b**) Parent Node Reselection; (**c**) Node Rewiring; and (**d**) $T_{2}$ Tree Expansion.

The principle of the RRT*-Connect algorithm is illustrated in Figure 2. Two random trees, $T_{1}$ and $T_{2}$, are initialized with $x_{init}$ and $x_{goal}$ as their root nodes, respectively. Taking the expansion of $T_{1}$ as an example, a random sampling point $x_{rand}$ is first generated from the free space. Then the node $x_{nearest}$, which has the smallest Euclidean distance to $x_{rand}$, is identified from $T_{1}$. Based on the $x_{nearest}$, a new node $x_{new}$ is generated by extending a step size *q* in the direction of $x_{rand}$. Subsequently, the neighboring nodes within a sampling radius $x_{near}$ from $x_{new}$ are identified from the tree to form the set $x_{near}$. A new parent node for $x_{new}$ is then selected from $x_{near}$ to minimize the path cost from the root node $x_{init}$ to $x_{new}$, after which the $x_{new}$ is added to the random tree. The algorithm then performs a rewiring step: it evaluates all the neighboring nodes of $x_{new}$ to check whether their path costs can be reduced via the $x_{new}$. If a lower-cost path is found, the parent node of the neighboring nodes is updated to the $x_{new}$. The $T_{2}$ tree is then expanded in the same pattern, extending towards the $x_{new}$ in $T_{1}$ to generate a new node $x_{new2}$. If no obstacle is encountered, the expansion continues. If an obstacle is encountered, the expansion of random tree $T_{2}$ is halted, and the trees $T_{1}$ and $T_{2}$ are swapped, proceeding to the next iteration. By alternately expanding random trees $T_{1}$ and $T_{2}$, the two trees grow towards each other until they successfully meet and connect.

The pseudocodes of the ‘RRT*-Connect’ algorithm and the extension function ‘Extend*(T, xrand)’ are illustrated in Algorithms 1 and 2. Initially, a sampling point $x_{rand}$ is generated via random sampling. Next, the ‘Extend*’ function is used to expand the random tree $T_{1}$ towards $x_{rand}$, creating a new node $x_{new}$ and returning the corresponding state value. If the return value of the ‘Extend*’ function is “Trapped”, the node $x_{new}$ is discarded, and the iteration proceeds to the next cycle; otherwise, the node $x_{new}$ is added to the random tree $T_{1}$.
**Algorithm 1 **RRT*-Connect  1:$T_{1}\leftarrow \left\{x_{init}\right\}$; $E_{1}\leftarrow \emptyset$; $T_{1}\leftarrow \left(V_{1},E_{1}\right)$  2:$V_{2}\leftarrow \left\{x_{goal}\right\}$; $E_{2}\leftarrow \emptyset$; $T_{2}\leftarrow \left(V_{2},E_{2}\right)$  3:**for**i=1 to *n* **do**  4:    $x_{rand}\leftarrow \mathrm{SampleFree()}$  5:    **if** ${\mathrm{Extend}}^{*}\left(T_{1},x_{rand}\right)\neq \mathrm{Trapped}$ **then**  6:      **if** ${\mathrm{Connect}}^{*}\left(T_{2},x_{new}\right)=\mathrm{Reached}$ **then**  7:        **return**$\left(T_{1},T_{2}\right)$  8:      **end if**  9:    **end if**10:  Swap($T_{1},T_{2}$)11:**end for**12:**return** (*T*_1_, *T*_2_)

**Algorithm 2 **Extend*($T,x_{rand}$)
  1:

$x_{nearest}\leftarrow \mathrm{Nearest}\left(T,x_{rand}\right)$

  2:

$x_{new}\leftarrow \mathrm{Steer}\left(x_{nearest},x_{new}\right)$

  3:**if** CollisionFree($x_{nearest},x_{new}$) **then**  4:   $V\leftarrow V\cup \{x_{new}\}$  5:   $E\leftarrow E\cup \{\left(x_{nearest},x_{new}\right)\}$  6:   $x_{near}\leftarrow \mathrm{Near}\left(T,x_{new},r\right)$  7:   $x_{min}\leftarrow \mathrm{ChooseParent}\left(T,x_{new},x_{near}\right)$  8:   $E\leftarrow E\cup \{\left(x_{new},x_{min}\right)\}$  9:   $T\leftarrow \mathrm{Rewire}(T,x_{new},x_{near})$10:   **if** $x_{new}=x$ **then**
11:     **return** Reached12:   **else**13:     **return** Advanced14:   **end if**15:
**else**
16:   **return** Trapped17:
**end if**



In the 3rd step of Algorithm 1 (Line 6), the ‘Connect*’ function is used to call the ‘Extend*’ function, expanding the random tree $T_{2}$ towards the new node $x_{new}$ in $T_{1}$. If the return value of the ‘Extend*’ function is “Advanced”, the tree $T_{2}$ continues to expand towards the $x_{new}$ until the return value is either “Trapped” or “Reached”. If the return value is “Trapped”, the expansion of the $T_{2}$ tree is halted, the two trees with the ‘Swap’ function are swapped, and the process enters the next iteration. If the return value is “Reached”, this indicates that the two trees are met and have been successfully connected, providing a collision-free path. Starting from the connection node in the $T_{1}$ tree, the parent nodes of each node are sequentially traced, and the node sequence is reversed to obtain the first half of the path. Similarly, starting from the connection node in $T_{2}$ tree, the parent nodes are sequentially traced, and the node sequence is arranged in order to obtain the second half of the path.

Although the RRT*-Connect algorithm can improve path-planning efficiency through bidirectional expansion and local optimization, its sampling process exhibits randomness and blindness, lacking guidance. Moreover, it samples and searches the entire space without constraints on the sampling region, leading to excessive exploration of invalid areas and low sampling efficiency. The algorithm employs a fixed step size, which also lacks flexibility and slows down the expansion process during the node expansion, making it more challenging to find optimal paths in complex, multi-obstacle and narrow environments. Besides, the paths generated by the algorithm often contain a huge amount of redundant nodes, resulting in higher path costs.

## 3. The Proposed IRRT*-Connect Algorithm

To address the limitations of the RRT*-Connect algorithm, this paper proposes an improved RRT*-Connect path-planning algorithm, i.e., IRRT*-Connect. The algorithm employs a heuristic sampling strategy which integrates the ellipsoidal subset constraints and goal-biased sampling. By sampling towards the target direction with a certain probability and constraining the sampling space within an ellipsoidal subset, the algorithm can iteratively compress the sampling space, thereby reducing the exploration of the invalid regions and enhancing the sampling efficiency in narrow passages. An adaptive step-size strategy is further introduced to dynamically adjust the step size based on the obstacle information. Combined with a node rejection strategy, the invalid nodes can be minimized, enabling faster convergence of the two trees. Further, the pruning strategy is applied to optimize the generated path by eliminating redundant nodes, thereby shortening the path length.

### 3.1. The Heuristic Sampling Strategy

We propose a heuristic sampling strategy that combines the ellipsoidal subset sampling and goal-biased sampling to improve the sampling efficiency during the path planning.

#### 3.1.1. Goal-Biased Sampling

The target bias probability α is defined with a range of $\left[0,1\right]$. During the sampling, a point is sampled towards the target direction with probability α, while with probability 1−α, the random sampling is performed in the free space. This strategy can maintain the randomness of the search while improving the search efficiency, expressed as

(1) $$x_{rand}=\left\{\begin{array}{cc} x_{goal} & \left(P<\alpha \right)\\ SampleFree() & \left(P\geq \alpha \right) \end{array}\right.$$

where *P* is the random number uniformly distributed in the range $\left[0,1\right]$. During each sampling iteration in the free space, the random tree can generate a random probability *P*. If P<α, the sampling point $x_{rand}$ is selected via target-biased sampling, where the random tree $T_{1}$ samples in the direction of the target point, and the random tree $T_{2}$ samples in the direction of the starting point. Otherwise, if P≥α, the sampling point $x_{rand}$ is generated randomly within the free space.

By introducing the goal-biased probability, the algorithm can maintain the randomness of the search while expanding towards the target region, thereby avoiding excessive exploration in areas far from the goal and accelerating path generation and convergence. The value of the goal-biased probability α is selected based on the complexity of the environment: in scenarios with more obstacles, a smaller α is chosen to prevent local optima, whereas in the environments with fewer obstacles, a larger α is adopted to enhance the convergence speed.

#### 3.1.2. Ellipsoidal Subset Sampling

**Figure 3:**
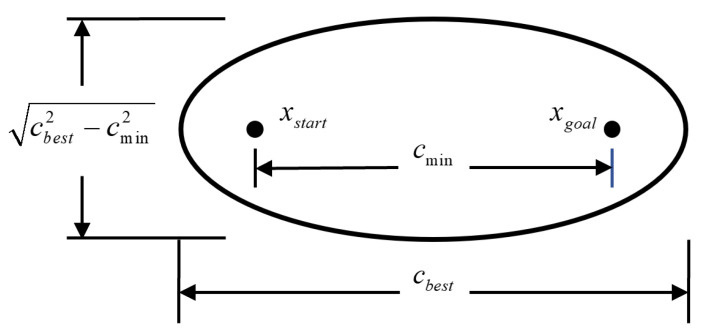
The ellipsoidal subset sampling.

The schematic diagram of ellipsoidal subset sampling is illustrated in Figure 3, where the starting point $x_{start}$ and the goal point $x_{goal}$ in the path are set as the two foci of the ellipse, $c_{min}$ represents the Euclidean distance between the starting point and the goal point, and $c_{best}$ denotes the length of the current shortest path. Specifically, $c_{best}$ is defined as the semi-major axis of the ellipsoid and $\sqrt{c_{best}^{2}-c_{min}^{2}}$ is defined as the semi-minor axis of the ellipsoid. By iteratively searching for the shorter paths, the ellipsoidal subset space can be reduced gradually.

To achieve the uniform sampling of the ellipsoidal subset, the samples are transformed from a sphere of radius *n* to the ellipsoidal subset, as follows: $x_{elipse}∼\mu \left(X_{elipse}\right)$,

(2) $$x_{ellipse}=Lx_{ball}+x_{center}$$

where $x_{center}=\left(x_{f1}+x_{f2}\right)/2$, $x_{ball}={\left\{x\in X|\|x\right\|}_{2}⩽1\}$, $x_{center}$ is the center of the hypersphere, $x_{ball}$ is the random sampling point from the sphere and *L* is the transformation matrix. The norm ${∥x∥}_{2}$ represents the Euclidean norm of *x* and $\left\{x_{f1},x_{f2}\right\}$ are the foci of the ellipse.

The transformation is calculated via the Cholesky decomposition of the hyperellipsoid matrix $S\in R^{n\times n}$, $S\equiv LL^{T}$, ${\left(x-x_{center}\right)}^{T}S\left(x-x_{center}\right)=I$ and the transformation matrix *L* can ensure that $x_{eplipse}$ maintains a uniform distribution. Therefore, the estimation of the subset of sampled points $x_{\hat{f}}$ can be achieved via the semi-major axis and semi-minor axis of the ellipse. The diagonal of the semi-major axis is given as $S=diag\left\{\frac{c_{best}^{2}}{4},\frac{c_{best}^{2}-c_{min}^{2}}{4},\ldots ,\frac{c_{best}^{2}-c_{min}^{2}}{4}\right\}$ and by performing the decomposition, we can obtain the matrix $L=diag\left\{\frac{c_{best}}{2},\frac{\sqrt{c_{best}^{2}-c_{min}^{2}}}{2},\ldots ,\frac{\sqrt{c_{best}^{2}-c_{min}^{2}}}{2}\right\}$.

To achieve the rotation of the hyperellipsoid into the world coordinate system, the rotation matrix *F* is computed based on the solution to Wahba’s problem,

(3) $$F=U\mathrm{diag}\left\{1,\cdots ,1,\det\left(U\right)\det\left(V\right)\right\}V^{T}$$

where det(·) denotes the determinant of a matrix. The matrices $U\in R^{n\times n}$ and $V\in R^{n\times n}$ are obtained from the unitary matrices of the Singular Value Decomposition (SVD) $U\Sigma V^{T}\equiv M$, Σ is a diagonal matrix with the absolute values of its diagonal entries equal to 1, matrix *M* is computed as the outer product of the first column of the identity matrix *I* and the semi-major axis length $a_{1}$ in the world coordinate system, i.e., $M=a_{1}I_{1}^{T}$, where $a_{1}=\left(x_{goal}-x_{start}\right)/∥x_{goal}-x_{start}∥$. Therefore, the subset of sampled points $x_{\hat{f}}$, obtained by transforming samples from a sphere of radius *n* via a transformation matrix, rotation matrix and translation process, is calculated as,

(4) $$x_{\hat{f}}=FLx_{ball}+x_{center}$$

Finally, the sampled points $x_{rand}$ within the ellipsoid are returned. The pseudocode of the ellipsoidal subset sampling algorithm is presented in Algorithm 3.
**Algorithm 3 **Informed_Sample($x_{start},x_{goal},c_{best}$)  1:**if** $c_{best}<\infty$**then**  2:   $c_{min}\leftarrow {∥x_{goal}-x_{start}∥}_{2}$  3:   $x_{center}\leftarrow \left(x_{start}+x_{goal}\right)/2$  4:   $c\leftarrow \mathrm{RotationToWorldFrame}(x_{start},x_{goal})$  5:   $r_{1}\leftarrow c_{best}/2$  6:   ${\left\{r_{i}\right\}}_{i=2,\ldots ,n}\leftarrow \sqrt{c_{best}^{2}-c_{min}^{2}/2}$  7:   $L\leftarrow \mathrm{diag}\{r_{1},r_{2},\ldots ,r_{n}\}$  8:   $x_{ball}\leftarrow \mathrm{SampleUnitBall}$  9:   $x_{rand}\leftarrow \left(FL\;x_{ball}+x_{center}\right)\cap X$10:**else**11:  $x_{rand}∼\mu \left(X\right)$12:**end if**13:**return** $x_{rand}$

### 3.2. The Adaptive Step Size

In the RRT*-Connect algorithm, the connection between a random point and its nearest point is extended in the direction of a fixed step size, and the extension strategy is defined as,

(5) $$x_{new}=x_{nearest}+q\frac{x_{nearest}-x_{rand}}{∥x_{nearest}-x_{rand}∥}$$

where *q* is the fixed step size. The usage of the fixed step size could limit the search ability in complex and narrow regions, and the expansion direction lacks the target bias, which would lead to slow convergence.

Here, an adaptive step size strategy is proposed and the new node expansion is redefined as

(6) $$x_{new}=x_{nearest}+k_{1}\frac{x_{nearest}-x_{rand}}{∥x_{nearest}-x_{rand}∥}+k_{2}\frac{x_{nearest}-x_{goal}}{∥x_{nearest}-x_{goal}∥}$$

where $k_{1}$ is the random step size and $k_{2}$ is the goal-directed step size. Through the experiments conducted with different upper and lower limits for $k_{1}$ and $k_{2}$, it is observed that an excessively large $k_{1}$ would cause the nodes to grow preferentially towards the random sampling points, thereby deviating from the target direction. Conversely, an excessively large $k_{2}$ would result in growth nodes being unable to bypass the obstacles. Therefore, the ranges for the step size $k_{1}$ and $k_{2}$ are set to [q,4q] and [0,4q], respectively, with initial values of $k_{1}=q$ and $k_{2}=0$.

From Equation (6), the new node extension direction is determined by a combination of the sampling point direction and the goal point direction, allowing more efficient navigation of the complex regions and faster convergence. The pseudocode of the adaptive expansion function is shown in Algorithm 4. The main process of the adaptive expansion function is described as follows:

(1) Determine which random tree grows and identify the extension direction. Then the ‘Nearest’ function is applied to return the nearest node $x_{nearest}$, and the ‘AdaptiveSteer’ function is applied to return the new node $x_{new}$.

(2) Perform collision detection via the method described in Section 2.1, check whether there is a collision between $x_{nearest}$ and $x_{new}$ along their connection, and return the corresponding state value *S*. If *S* returned by the current extension function is “Advanced”, it indicates that the extension is successful, the robotic arm is in a collision-free state, and the new node of the current extension function is not connected to the other random tree. In the next extension, the ‘AdaptiveStep’ function is applied to increase $k_{1}$ and $k_{2}$ by q/2 and *q*, respectively. If *S* returned by the next adaptive extension function is still “Advanced”, then $k_{1}$ and $k_{2}$ continue to increase by q/2 and *q*, respectively. $k_{2}$ will be greater than $k_{1}$ to improve the extension towards the target direction until $k_{1}$ and $k_{2}$ reach the upper limit of the threshold.

(3) If *S* is returned, the next ‘AdaptiveExtend’ function is “Trapped”, indicating that a collision occurs between the new node extended by the ‘AdaptiveExtend’ function and the nearest node. In the next extension, the random tree will extend according to the initial values; $k_{2}$ will be less than $k_{1}$ so as to extend towards the random sampling point to avoid the obstacles.
**Algorithm 4 **AdaptiveExtend($T,x_{rand},k_{1},k_{2}$)  1:$x_{target}\leftarrow x_{goal}$  2:**if** $T\neq T_{1}$  **then**  3:   $x_{target}\leftarrow x_{init}$  4:**end if**  5:$x_{nearest}\leftarrow \mathrm{Nearest}\left(T,x_{rand}\right)$  6:$k_{1},k_{2}\leftarrow \mathrm{Adaptivestep}\left(S\right)$  7:$x_{new}\leftarrow \mathrm{AdaptiveSteer}\left(x_{nearest},x_{rand},x_{target},k_{1},k_{2}\right)$  8:**if** CollisionFree($x_{nearest},x_{new}$) **then**  9:    **if** $x_{new}=x_{rand}$ **then**
10:     **return** $x_{new},S\leftarrow \mathrm{Reached}$11:   **else**12:     **return** $x_{new},S\leftarrow \mathrm{Advanced}$13:   **end if**14:**else**15:**   return** $x_{new},S\leftarrow \mathrm{Trapped}$16:**end if**

### 3.3. The Node Rejection

After a new node is generated via the adaptive extension function, it is necessary to evaluate whether the node can effectively reduce the path cost. Therefore, we propose a node rejection strategy,

(7) $$∥x-x_{init}∥+∥x_{goal}-x∥>\;c_{best}$$

where the Euclidean distance $∥x-x_{init}∥$ and $∥x_{goal}-x∥$ are calculated to estimate the cost to the node and the cost from the node to the goal point. If the estimated total cost of the node exceeds the best path cost $c_{best}$, it indicates that the node will not effectively reduce the path cost and it is removed from the tree. This approach can result in fewer nodes in the random tree and fewer extensions with reduced computational costs and higher planning efficiency [33].

### 3.4. The Pruning Optimization

In order to efficiently delete these redundant nodes, this paper proposes a pruning optimization strategy based on the triangle inequality to optimize the generated path.

**Figure 4:**
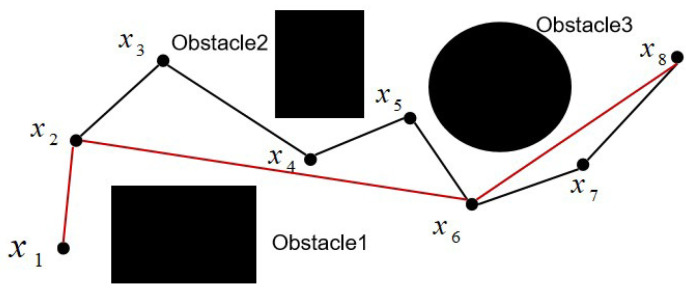
Illustration of the pruning optimization.

As displayed in Figure 4, the robotic arm is required to move from point $x_{1}$ to point $x_{8}$ while avoiding three obstacles. The black path, $x_{1}-x_{2}-x_{3}-x_{4}-x_{5}-x_{6}-x_{7}-x_{8}$, is the path generated by the algorithm. However, under the assumption that the robotic arm does not collide, it can move directly from $x_{2}$ to $x_{6}$, then from $x_{6}$ to $x_{8}$. Therefore, the points $x_{3}$, $x_{4}$, $x_{5}$, and $x_{7}$ are redundant. Using the principle of pruning based on the triangle inequality, the redundant nodes are removed from the black path, resulting in the red path $x_{1}-x_{2}-x_{6}-x_{8}$, having a shorter distance.

**Figure 5:**
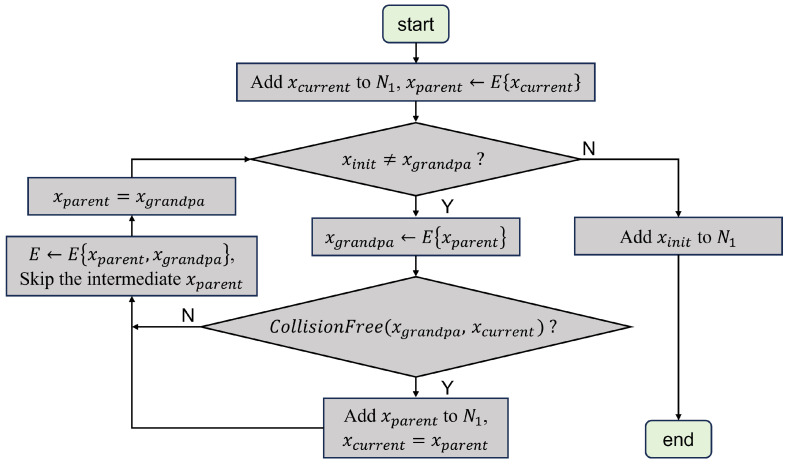
The flowchart of the branch pruning optimization process.

The path point set generated by RRT*-connect is the union of the path point sets of the two random trees, $T_{1}$ and $T_{2}$. Taking the optimization of the random tree $T_{1}$ as an example, $x_{init}$ is the root node of the random tree $T_{1}$ and the connected node between the two trees is the current node $x_{current}$. Figure 5 displays the specific branch pruning optimization process.

(1) Initialization: Add $x_{current}$ to the empty set $N_{1}$, then obtain the parent node $x_{parent}$ of the current node via the edge structure.

(2) Check Grandparent Node: If the grandparent node $x_{grandpa}$ (the parent of $x_{parent}$) is not the root node $x_{init}$, proceed to the next step 3.

(3) Edge Structure Update: Use the edge structure to obtain the grandparent node $x_{grandpa}$.

(4) Collision Check: Determine whether the robotic arm collides with an obstacle along the connection between the current node $x_{current}$ and the grandparent node $x_{grandpa}$. If no collision occurs, the parent of the current node is updated as the grandparent node $x_{grandpa}$, the edge structure is updated, and the intermediate node $x_{parent}$ is skipped. If a collision occurs, the $x_{parent}$ is added to the set $N_{1}$ and the current node $x_{current}$ is updated as the $x_{parent}$.

(5) Repeat Process: Continue to repeat steps 2 to 4 until the grandparent node backtracks to the root node $x_{init}$ (i.e., $x_{grandpa}=x_{init}$), add the root node $x_{init}$ to the set $N_{1}$ and end the loop. The optimized path set $N_{1}$ is returned.

The pruning optimization process of the random tree $T_{2}$ is similar to that of $T_{1}$. The only difference is that the root node $x_{init}$ is replaced with the goal node $x_{goal}$, while the same steps 1–5 are implemented.

### 3.5. The Process of the Proposed IRRT*-Connect Algorithm

The proposed IRRT*-Connect algorithm utilizes an ellipsoidal subset constraint and a target-bias heuristic sampling strategy to reduce the exploration of the invalid space, so as to improve the sampling efficiency and lower the time costs. It also employs an adaptive step-size strategy to dynamically adjust the step size, enabling faster convergence of the two trees. By integrating a node rejection strategy, the expansion of the invalid nodes can be minimized. Furthermore, a pruning strategy is applied to optimize the generated path with shorter path length. The pseudocode of the algorithm is shown in Algorithm 5.
**Algorithm 5 **IRRT*-Connect  1:$V_{1}\leftarrow \left\{x_{init}\right\}$; $E_{1}\leftarrow \emptyset$; $T_{1}\leftarrow \left(V_{1},E_{1}\right)$  2:$V_{2}\leftarrow \left\{x_{goal}\right\}$; $E_{2}\leftarrow \emptyset$; $T_{2}\leftarrow \left(V_{2},E_{2}\right)$  3:$x_{solin}\leftarrow \emptyset$; $c_{\mathrm{best}}\leftarrow \infty$; count←0; $k_{1}=q,k_{2}=0$  4:**for** i=1 to *n* **do**  5:   $c_{best}\leftarrow \mathrm{ShortestPathLength}\left(x_{solin}\right)$  6:   $x_{rand}\leftarrow \mathrm{ImprovedSampleFree}\left(x_{init},x_{goal},c_{best},\alpha \right)$  7:   $x_{new},S\leftarrow \mathrm{AdaptiveExtend}\left(T_{1},x_{rand},k_{1},k_{2}\right)$  8:   **if** S≠Trapped **and** $\mathrm{NodeReject}(x_{init},x_{goal},x_{new},c_{best})=\mathrm{False}\mathrm{then}$  9:     $V\leftarrow V\cup \{x_{new}\}$10:     $E\leftarrow E\cup \{\left(x_{nearest},x_{new}\right)\}$11:   **end if**12:   $L_{near}\leftarrow \mathrm{GetNearbyVertices}\left(V_{1},x_{best},x_{\mathrm{new}}\right)$13:   $x_{min}\leftarrow \mathrm{ChooseParent}\left(V_{1},x_{new},L_{near}\right)$14:   **if** $x_{\min}\neq \emptyset$ **then**15:     $E_{1}\leftarrow E_{1}\cup \left\{x_{new},x_{min}\right\}$16:     $V_{1}\leftarrow \mathrm{Rewire}\left(V_{1},L_{near},x_{new}\right)$17:   **end if**18:   **if** S≠Trapped **then**19:     $x_{new2},\mathrm{flag}\leftarrow {\mathrm{ImprovedConnect}}^{*}\left(V_{2},x_{new}\right)$20:     **if** flag=Reached **then**21:        $x_{solin},count\leftarrow \mathrm{OptimizeTrees}\left(T_{1},T_{2},x_{new2}\right)$22:     **end if**23:   **end if**24:   SwapTrees($T_{1},T_{2}$)25:   **if** count≥4 **then**26:     $T_{1},T_{2}=\left(V,E\right)$27:   **end if**28:**end for**29:**return** $\left(T_{1},T_{2}\right)$

(1) Initialization: Lines 1–2 of the algorithm are the same as in Algorithm 1. Let $x_{soln}$ be the set of all path solutions, $c_{best}$ be the current shortest path length, and count be the number of found path solutions. Initially, before a path solution is found, $x_{soln}$ is an empty set, $c_{best}$ is infinity and count is zero.

(2) Shortest Path Length Calculation: Use the ‘ShortestPathLength’ function to compute the shortest path length $c_{best}$ from the set $x_{soln}$.

(3) Sampling and Extension: Use the ‘ImprovedSamplefree’ function to generate a random sample point $x_{rand}$ in the free space. Then, use the ‘AdaptiveExtend’ function to extend the tree and generate a new node $x_{new}$, and return the state value *S*.

(4) State Value and Path Cost Evaluation: Check *S* and calculate the total estimated path cost for the $x_{new}$. If *S* is not “Trapped” and the total estimated path length of $x_{new}$ is less than $c_{best}$, add $x_{new}$ to the tree and set the parent of $x_{new}$ to be $x_{nearest}$. Otherwise, swap the random trees $T_{1}$ and $T_{2}$, re-sample, and return to step 2.

(5) Find Neighboring Nodes and Parent Reselection: Use the ‘GetSortedList’ function to find the neighboring nodes $x_{near}$ within a spherical region centered around $x_{new}$ in the random tree. Sort the filled set $x_{near}$ and return the list $L_{near}$ ordered by the cost function in ascending order. Next, use the ‘ChooseParent’ process to traverse the sorted list, return the optimal parent node $x_{min}$ that connects $x_{new}$ and $x_{init}$ in the free space. If such a node exists, set $x_{min}$ as the optimal parent of $x_{new}$, and perform the rewiring process.

(6) Tree Connection and Path Optimization: If *S* is not ’Trapped’, the ‘ImprovedConnect’ function is used to call the ‘AdaptiveExtend’ function to extend the random tree $T_{2}$ towards the new node $x_{new}$ in tree $T_{1}$ and attempt to connect the trees. If the trees fail to connect, the function returns “Trapped”, $T_{1}$ and $T_{2}$ are swapped, and $T_{2}$ is prioritized for expansion in the next iteration. If the connection is successful, “Reached” is returned, indicating that the two trees are connected and a path is found. Then path optimization is performed which returns the set of path solutions $x_{soln}$, and the number of path solutions found is recorded as ‘count‘. If $‘count^{\prime }$ is less than 4, it continues iterating and performs steps 2–6. If ‘count‘ exceeds 4, the optimal path is returned. This process can iteratively refine the path solutions by expanding the trees, checking for collisions, and optimizing the path until a high-quality solution is found.

### 3.6. Algorithm Time–Space Complexity Analysis

In this study, the time cost of the IRRT*-Connect algorithm is primarily affected by steps such as nearest node search and collision detection. In each iteration, the algorithm performs collision detection for newly generated nodes, resulting in a time complexity of O(N+M) per iteration, *N* represents the number of nodes in the path-planning tree and *M* denotes the number of obstacles. Consequently, the overall time complexity of the algorithm is O(T·(N+M)), where *T* represents the number of iterations. By employing spatial partitioning data structures (e.g., R-tree), the time complexity of the nearest node search can be optimized to O(logN), significantly reducing the overall time complexity.

In terms of the spatial complexity, the IRRT*-Connect algorithm must maintain the nodes of the path-planning tree and a cache of the obstacle information. This can result in a spatial complexity of O(N+M). The space requirements dynamically change during the algorithm execution but are generally linearly related to the scale of the data being processed.

### 3.7. Path Smoothing

The path generated by the IRRT*-Connect algorithm has fewer points and shorter length. However, at certain path points, sharp corners can cause vibrations in the robotic arm, potentially damaging its mechanical components. Therefore, it is necessary to smooth the generated path. B-spline curves, known for their flexibility, continuity and differentiability, are widely used in robotic arm path planning. In this paper, we use a cubic non-uniform B-spline interpolation algorithm to smooth the path and generate an executable trajectory for the robotic arm. Let the path generated by IRRT*-Connect be denoted as *x*, with $x_{i}$ (i=1,2,...,n) being the path points of *x*. The cubic non-uniform B-spline interpolation is applied to fit the path *x*. The mathematical expression of a B-spline curve is written as,

(8) $$p\left(u\right)=\sum_{i=0}^{n}d_{i}N_{i,k}\left(u\right)$$

where $d_{i}$ (i=1,2,...,n) is the control point, and $N_{i,k}\left(u\right)$ is the basis function of the *k* times of the B-spline.

For the cubic non-uniform B-spline curve, assuming that the length of each edge of the control polygon is $l_{i}=\left|x_{i}-x_{i-1}\right|$ (i=1,2,...,n), and the total length is $L=\sum_{i=1}^{n}l_{i}$. Based on the chord-length parameterized node vector, the node vector is obtained,

(9) $$U=\left[0,0,0,0,\frac{l_{1}+l_{2}}{L},\frac{l_{1}+l_{2}+l_{3}}{L},L,\frac{\sum_{j=1}^{n-2}l_{j}}{L},1,1,1,1\right]$$

Subsequently, the B-spline basis functions are computed via the recursive function,

(10) $$\left\{\begin{array}{c} N_{i,0}\left(u\right)=\left\{\begin{array}{c} 1,u_{i}\leq u\leq u_{i+1}\\ 0,others \end{array}\right.\\ N_{i,k}=\frac{u-u_{i}}{u_{i+k}-u_{i}}N_{i,k-1}\left(u\right)+\frac{u_{i+k+1}-u}{u_{i+k+1}-u_{i+1}}N_{i+1,k-1}\left(u\right)\\ \frac{0}{0}=0;0\times \infty =0 \end{array}\right.$$

The cubic non-uniform B-spline function for reverse-solving control points is expressed as,

(11) $$\left[\begin{array}{cccc} N_{1,3}\left(u_{3}\right) & N_{2,3}\left(u_{3}\right) & \; & N_{0,3}\left(u_{3}\right)\\ N_{1,3}\left(u_{4}\right) & N_{2,3}\left(u_{4}\right) & N_{3,3}\left(u_{4}\right) & \\ \ddots & \ddots & \ddots & \\ \; & N_{n-2,3}\left(u_{n+2}\right) & N_{n-1,3}\left(u_{n+2}\right) & N_{n,3}\left(u_{n+2}\right)\\ N_{n+1,3}\left(u_{n+3}\right) & \; & N_{n-1,3}\left(u_{n+3}\right) & N_{n,3}\left(u_{n+3}\right) \end{array}\right]\left[\begin{array}{c} d_{1}\\ d_{2}\\ \vdots \\ d_{n-1}\\ d_{n} \end{array}\right]=\left[\begin{array}{c} x_{0}\\ x_{1}\\ \vdots \\ x_{n-2}\\ x_{n-1} \end{array}\right]$$

Since all the elements in the coefficient matrix are values of the B-spline basis functions, they are solely related to the knot vector, so Equation (11) can be simplified as Equation (12),

(12) $$\left[\begin{array}{ccccc} b_{1} & c_{1} & \; & & a_{1}\\ a_{2} & b_{2} & c_{2} & \; & \\ \; & \ddots & \ddots & \ddots & \\ \; & \; & a_{n-1} & b_{n-1} & c_{n-1}\\ c_{n} & \; & & a_{n} & b_{n} \end{array}\right]$$

(13) $$\left\{\begin{array}{c} a_{i}=\frac{{\left(\Delta_{i+2}\right)}^{2}}{\Delta_{i}+\Delta_{i+1}+\Delta_{i+2}}\\ b_{i}=\frac{\left(\Delta_{i}+\Delta_{i+1}\right)\Delta_{i+2}}{\Delta_{i}+\Delta_{i+1}+\Delta_{i+2}}+\frac{\Delta_{i+1}\left(\Delta_{i+2}+\Delta_{i+3}\right)}{\Delta_{i+1}+\Delta_{i+2}+\Delta_{i+3}}\\ c_{i}=\frac{{\left(\Delta_{i+1}\right)}^{2}}{\Delta_{i+1}+\Delta_{i+2}+\Delta_{i+3}}\\ e_{i}=\left(\Delta_{i+1}+\Delta_{i+2}\right)x_{i-1} \end{array}\right.$$

**Figure 6:**
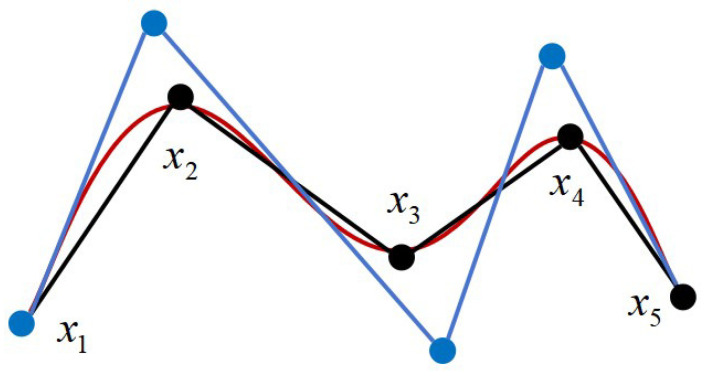
The cubic inhomogeneous B-spline interpolation.

By solving Equation (11), all the control points can be determined. Subsequently, with the knot vector, the control points are obtained, and the interpolation adjustment of Equation (8) is performed for the path points, resulting in a smooth trajectory. As illustrated in Figure 6, the black path segment $x_{1}-x_{2}-x_{3}-x_{4}-x_{5}$ represents the trajectory planned by the IRRT*-Connect algorithm. The blue points denote the computed control points, while the red curve is the trajectory obtained through cubic non-uniform B-spline interpolation.

## 4. Experiment and Result Analysis

To validate the effectiveness and reliability of the proposed algorithm, a series of experiments are conducted. First, simulations of 2D and 3D static obstacle maps are performed via the Python programming language, followed by robotic arm obstacle avoidance simulations in the ROS environment. Subsequently, a physical experimental platform is established in a static multi-obstacle environment to verify the practical performance of the algorithm. All experiments are conducted under the same hardware configuration: an AMD Ryzen 7 5800H CPU (3.20 GHz), 16 GB of RAM, and an NVIDIA GTX 1650 GPU.

In the 2D and 3D static obstacle map experimental environments constructed in Python, each algorithm is evaluated under identical parameter settings, with a step size *q* of 4 mm, a search radius *r* of 5 mm, a maximum iteration count of 2000, and a goal-biased probability of 0.1. The search radius *r* denotes the range for seeking new nodes, which is employed for local optimization. However, an excessively large value of *r* will increase the computational burden. Therefore, it is advisable to set *r* slightly larger than the step size. The parameter settings presented in this study are mainly determined via extensive experiments and human experience. To account for the stochastic nature of the sampling-based algorithms, each experiment is repeated 100 times, and the final results are averaged to ensure reliability and consistency.

### 4.1. The 2D Static Multi-Obstacle Narrow Environment Simulation

**Figure 7:**
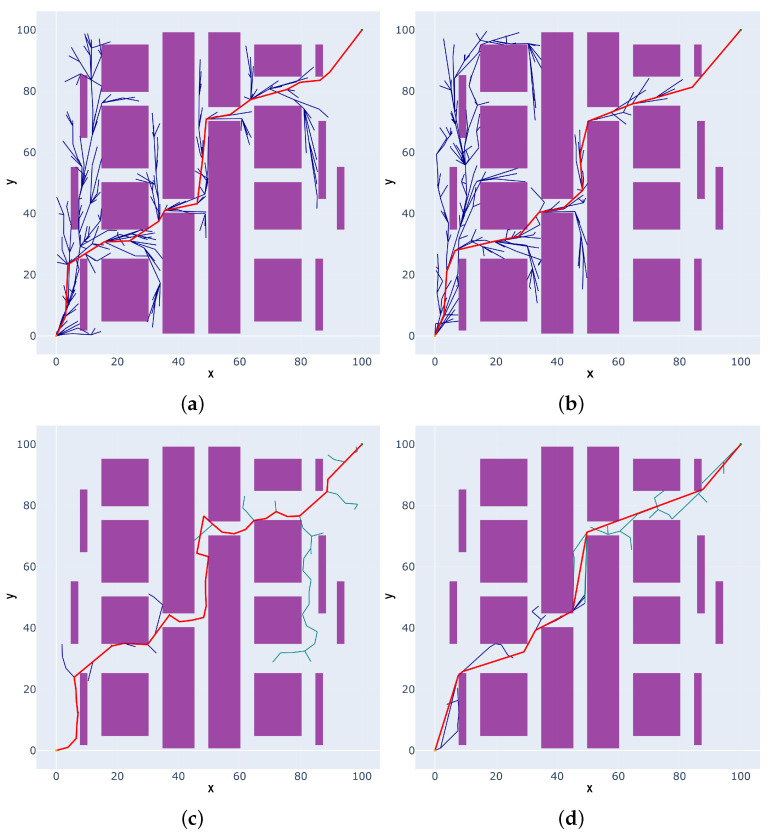
The comparison results of 2D static multi-obstacle narrow space path-planning algorithms in Scenario I: (**a**) RRT*; (**b**) Informed-RRT*; (**c**) RRT*-Connect; and (**d**) IRRT*-Connect.

In a Python environment, a 2D static multi-obstacle narrow environment is constructed for Scenario 1, as illustrated in Figure 7. The map size is 100 mm × 100 mm, with a minimum distance of 5 mm between narrow passages. The purple rectangular objects represent the obstacles, the yellow circle denotes the start point at coordinates (0, 0), and the green circle indicates the goal point at coordinates (100, 100). The blue curve represents the path randomly expanded by the planning algorithm from the start point, the green curve represents the path expanded from the goal point and the red curve denotes the final generated path.

The proposed algorithm (IRRT*-Connect) is compared with the RRT*, RRT*-Connect, and Informed-RRT* algorithms in a 2D simulation. From Figure 7, it can be observed that the RRT* and Informed-RRT* exhibit more random tree branches, while the RRT*-Connect has fewer branches but longer path length. In contrast, the proposed IRRT*-Connect algorithm exhibits a clearer and more focused search direction with fewer branches and a shorter path. The results are listed in Table 1 and the proposed algorithm achieves reductions in the path cost by 4.22%, 3.04%, and 15.16% compared to RRT*, Informed-RRT*, and RRT*-Connect, respectively, and reductions in time cost by 66.67%, 71.43%, and 42.86%. Therefore, the proposed algorithm demonstrates advantages in both path cost and time cost over the RRT*, Informed-RRT*, and RRT*-Connect algorithms.

**Figure 8:**
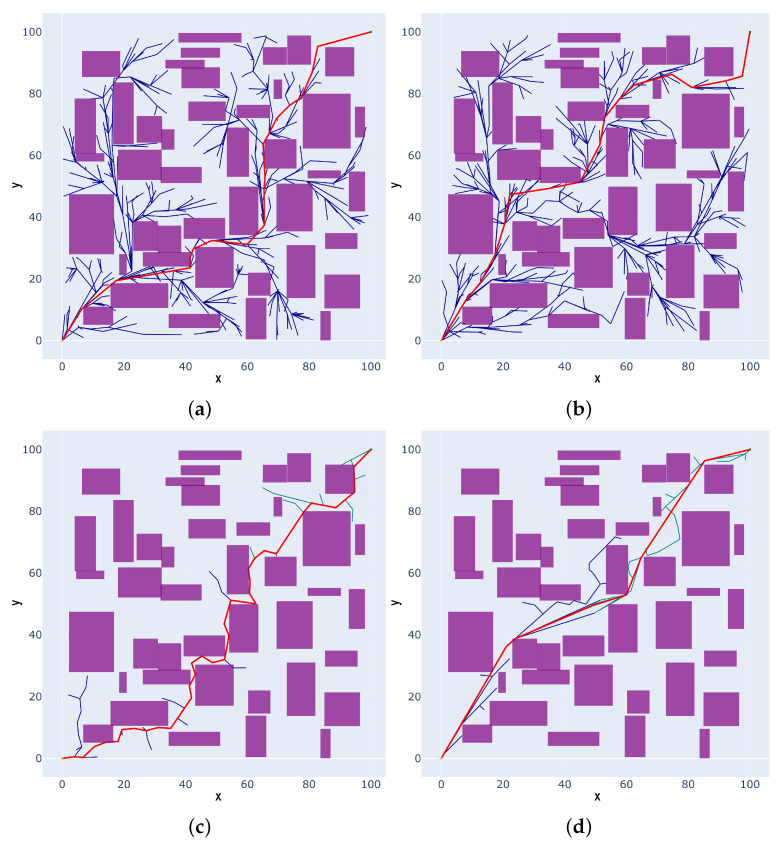
The comparison results of 2D static multi-obstacle narrow space path-planning algorithms in Scenario II: (**a**) RRT*; (**b**) Informed-RRT*; (**c**) RRT*-Connect; and (**d**) IRRT*-Connect.

In Scenario 2, a 2D static narrow environment with multiple obstacles is used, as depicted in Figure 8. From the empirical data in Table 2, it can be observed that the RRT* algorithm achieves an average path length of 163.39 mm and an average planning time of 0.56 s. In contrast, while the Informed-RRT* algorithm reduces the path cost, the average planning time increases to 0.76 s. The RRT*-Connect algorithm with the bidirectional search strategy can significantly optimize the planning time, reducing the average planning time to 0.27 s. However, the path length increases to 186.05mm. The proposed IRRT*-Connect algorithm demonstrates the best performance across all metrics, achieving an average path length of 158.48 mm and an average planning time of 0.18 s. Compared to the RRT*, Informed-RRT*, and RRT*-Connect algorithms, the IRRT*-Connect algorithm can reduce the path cost by 3.01%, 2.34%, and 14.82%, respectively, and decrease the planning time by 67.86%, 76.32%, and 33.33%, respectively.

### 4.2. The 3D Static Multi-Obstacle Narrow Environment Simulation

**Figure 9:**
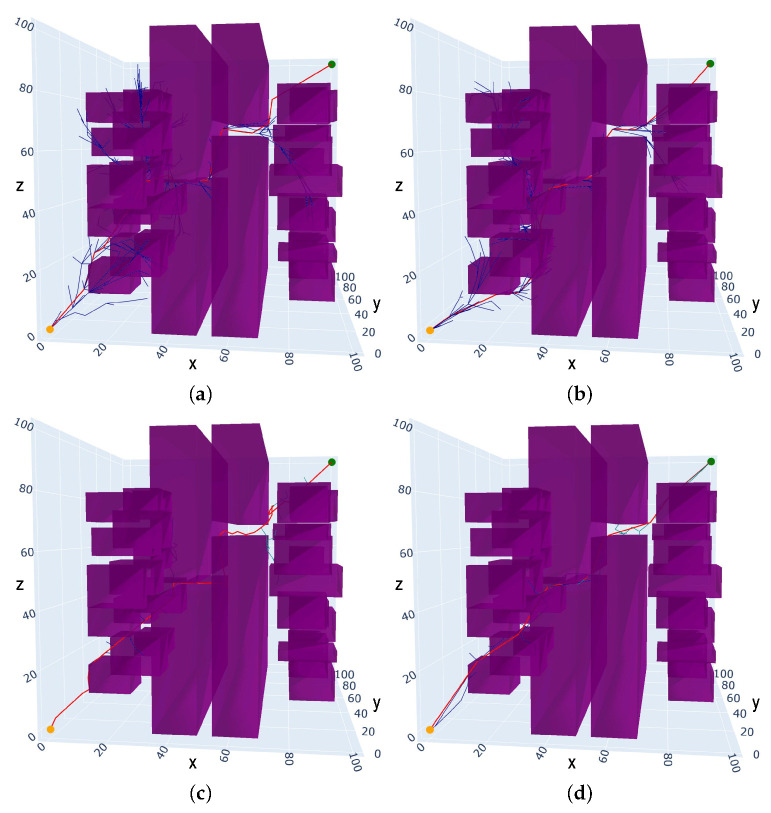
The comparison results of 3D static multi-obstacle narrow space path-planning algorithms in Scenario III: (**a**) RRT*; (**b**) Informed-RRT*; (**c**) RRT*-Connect; and (**d**) IRRT*-Connect.

In Python, a 3D static multi-obstacle narrow environment (Scenario III) is constructed, as shown in Figure 9. The map size is set to 100 mm × 100 mm × 100 mm to test the adaptability of the proposed algorithm in a complex narrow passage of a 3D static environment. The purple rectangular prisms represent the obstacles, the yellow circle represents the start point located at (0,0,0), the green circle represents the target point located at (100,100,100), and the minimum clearance between the narrow passages is 5mm.

The proposed algorithm (IRRT*-Connect) is compared with the RRT*, RRT*-Connect and Informed-RRT* algorithms in the 3D simulation. From Figure 9, the random trees generated by the RRT* and Informed-RRT* algorithms have a larger number of branches, while the random tree generated by the RRT*-Connect algorithm has fewer branches. However, the path generated by RRT*-Connect is more convoluted. The experiment results are listed in Table 3. In the 3D environment with multiple obstacles and narrow spaces, the Informed-RRT* algorithm can reduce the path cost by 9.67% and 1.36% compared to RRT*-Connect and RRT*, while the time cost is increased by 55.88% and 14.71%, respectively. The proposed algorithm can achieve a reduction in path cost by 11.42%, 10.20%, and 18.88%, respectively, and a reduction in the time cost by 70.69%, 75.00%, and 43.33% compared to the RRT*, Informed-RRT*, and RRT*-Connect algorithms.

**Figure 10:**
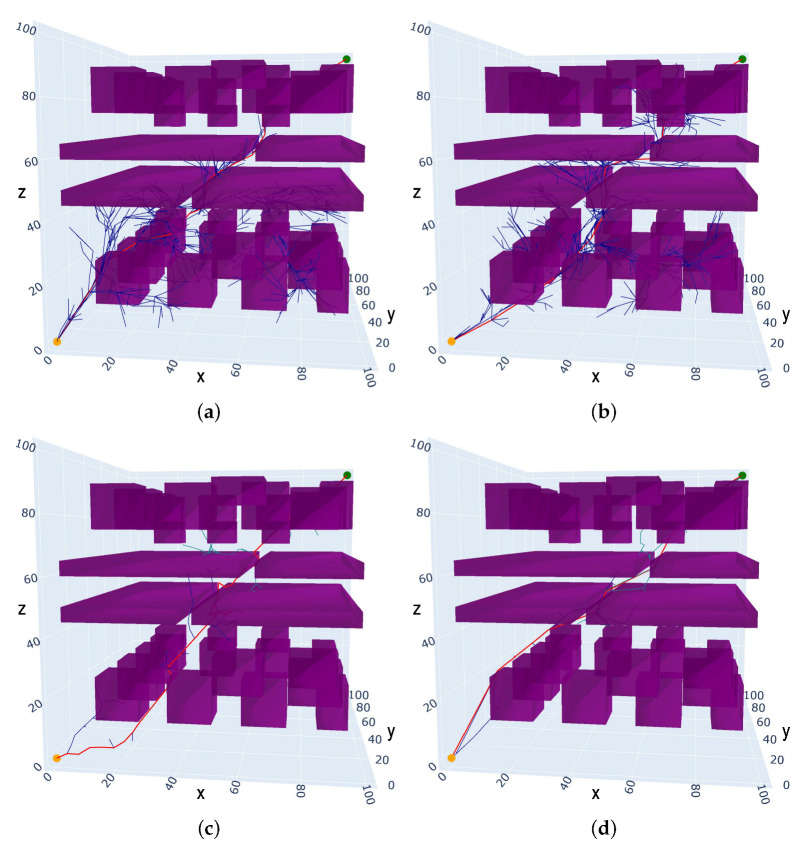
The comparison results of 3D static multi-obstacle narrow space path-planning algorithms in Scenario IV: (**a**) RRT*; (**b**) Informed-RRT*; (**c**) RRT*-Connect; and (**d**) IRRT*-Connect.

In Scenario IV, a 3D static narrow environment with multiple obstacles is depicted in Figure 10, with the starting position and goal position identical to those in Scenario IV. From the experimental data in Table 4, it can be observed that the RRT* algorithm achieves an average path length of 210.28 mm and an average planning time of 1.12 s. Compared to Scenario iiI, both the path cost and time cost have increased. The Informed-RRT* algorithm exhibits a higher average planning time of 1.26 s but achieves a lower path cost than that of the RRT*. The RRT*-Connect algorithm maintains its advantage in time cost but spends a higher path cost compared to the other algorithms. The IRRT*-Connect algorithm outperforms other algorithms across all metrics, achieving an average path length of 181.78 mm and an average planning time of 0.18 s. Compared to the RRT*, Informed-RRT*, and RRT*-Connect algorithms, the IRRT*-Connect algorithm can reduce the path cost by 13.55%, 11.56%, and 15.38%, respectively, and can reduce the time cost by 83.93%, 85.71%, and 35.71%, respectively.

Through employing the elliptical subset constraint and the target bias heuristic sampling strategy in these experiments, it can be concluded that the proposed IRRT*-Connect algorithm can achieve more accurate and effective sampling directions, with reduced sampling space and minimized exploration of the invalid areas. The adaptive step-size method can dynamically adjust the step size, allowing for faster convergence of the two trees. Additionally, the node selection strategy can reduce the expansion of the invalid nodes, and the pruning strategy based on the triangle inequality can optimize the generated path. Therefore, the proposed IRRT*-Connect algorithm demonstrates higher planning efficiency and lower path costs.

### 4.3. The Field Experiment of the Robotic Arm

To evaluate the effectiveness of the proposed IRRT*-Connect algorithm in practical applications, experiments are conducted within the Robot Operating System (ROS) framework using the UR5 robotic arm. Initially, a 3D model of the robotic arm is created via the URDF file. Subsequently, the MoveIt motion planning framework is utilized to configure and generate the necessary launch files for trajectory planning, and the 3D model is visualized in rviz. The proposed algorithm is integrated into the OMPL motion planning library to control the robotic arm for obstacle avoidance through the MoveIt motion planning framework.

**Figure 11:**
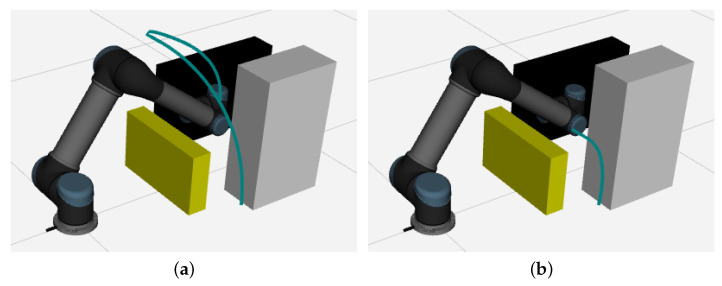
The robotic arm motion obstacle avoidance process: (**a**) RRT*-Connect; (**b**) IRRT*-Connect.

**Figure 12:**
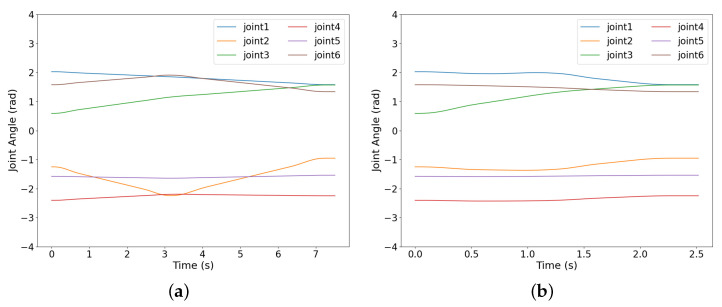
The curves of each joint changing over time: (**a**) RRT*-Connect; (**b**) IRRT*-Connect.

A static obstacle simulation environment for the robotic arm obstacle avoidance (Scenario V) is set up in rviz, as illustrated in Figure 11, where yellow, black, and gray rectangular prisms represent the static obstacles. The blue curve depicts the trajectory of the UR5 robotic arm’s end-effector. Figure 12 illustrates the joint motion curves during the obstacle avoidance process. The initial joint angles of the robotic arm are set to $\left[34^{\circ },-71.46^{\circ },116.19^{\circ },-137.54^{\circ },-90.23^{\circ },90.50^{\circ }\right]$ (in degrees), and the target joint angles are set to $\left[90^{\circ },-54.37^{\circ },90.66^{\circ },-128.66^{\circ },-88.01^{\circ },76.83^{\circ }\right]$.

It can be seen in Figure 11 and Figure 12 that no collision occurs between the robotic arm and the obstacles. The IRRT*-Connect algorithm, in conjunction with cubic non-uniform B-spline interpolation, can generate a sufficiently smooth trajectory, enabling stable motion of the robotic arm without any jitter. Compared to RRT*-Connect, the proposed algorithm yields a shorter motion trajectory with no sharp variations or discontinuities.

**Figure 13:**
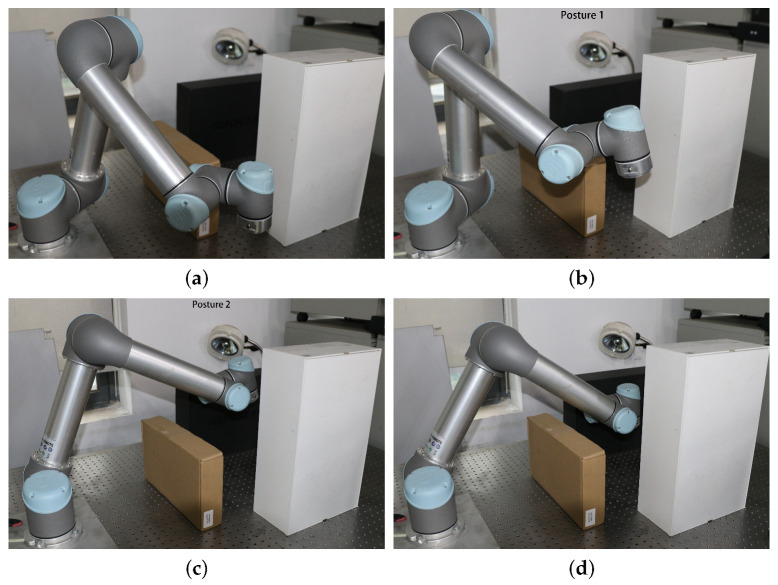
The process of the robotic arm motion obstacle avoidance: (**a**) starting position; (**b**) obstacle avoidance posture 1; (**c**) obstacle avoidance posture 2; and (**d**) end pose.

Further, to verify the performance of obstacle avoidance of the real arm equipped with the proposed algorithm, a real-world environment with multiple static obstacles in a narrow space was set up, as seen in Figure 13. The sizes and spatial positions of the white, black, and yellow rectangular prism obstacles are consistent in the rviz simulation environment. With the integration of the MoveIt motion planning framework with the ROS, the real-time control of the UR5 robotic arm and its interaction with the environment can be achieved. Within this framework, the robotic arm obstacle avoidance experiments are precisely synchronized and scheduled via the ROS topics and services. Specifically, the computer is connected to the UR5 robotic arm control cabinet via the Ethernet. Leveraging the MoveIt framework, the IRRT*-Connect algorithm is invoked to perform motion planning. The path information, including the planning time, trajectory length, and the joint angle configurations corresponding to each waypoint, is transmitted to the control cabinet. This enables the physical UR5 manipulator to execute the obstacle avoidance motions which precisely follow the planned trajectory in the simulation environment.

The initial configuration of the manipulator is shown in Figure 13a. During the execution, the manipulator navigates through a narrow passage formed by a yellow and a white obstacle, avoiding collisions as it enters the enclosed space surrounded by three obstacles. It then proceeds downward and ultimately reaches the target position shown in Figure 13d. Two key obstacle-avoidance configurations, ‘posture 1’ and ‘posture 2’ during the motion are captured in Figure 13b,c. Throughout the entire trajectory, the manipulator can successfully avoid all obstacles and operates smoothly without any collision.

In order to validate the feasibility and superiority of the algorithm in the real-world experiments, twenty simulation trials have been conducted, and the results are averaged and listed in Table 5.

The data presented in Table 5 demonstrate that the IRRT*-Connect algorithm can significantly outperform the RRT*-Connect algorithm, achieving a 34.51% reduction in the planning time and a 14.31% decrease in the path cost, while also improving the task success rate by 14%. These experimental results indicate that the IRRT*-Connect exhibits superior performance in the physical experiments, effectively meeting the motion planning requirements of the robotic manipulators. Further, it can substantially reduce both the planning time and the path cost, making it a highly efficient solution for the practical implementations.

## 5. Conclusions

This paper proposes the IRRT*-Connect path planning algorithm to address the limitations of the traditional RRT*-Connect algorithm in static multi-obstacle and narrow-space environments, including low sampling efficiency, extended computation time and high path cost. The proposed algorithm can improve the sampling efficiency by employing the dual-tree expansion combined with heuristic sampling techniques, such as ellipsoid subset sampling and goal-biased sampling, which can guide the sampling process towards the target with a certain probability while reducing the exploration in the invalid regions. Further, an adaptive dynamic step-size method is introduced to adjust the step size based on the obstacle information, and a node rejection strategy is applied to accelerate the convergence, while the path-pruning strategy is further used to shorten the planned trajectory. A cubic non-uniform B-spline interpolation algorithm is employed to smooth the path during robotic arm motion, preventing oscillations at sharp turns and reducing mechanical wear.

The effectiveness and superiority of the proposed algorithm are validated via simulations in both 2D and 3D static obstacle maps, as well as ROS-based simulations. Compared to the RRT*-Connect algorithm, the proposed approach can significantly reduce the planning time and shorten the path length. In practical experiments, the robotic arm is able to complete the obstacle avoidance tasks swiftly and smoothly. Therefore, the proposed method is well suited for applications such as robotic arm handling, assembly, and painting in static multi-obstacle environments, enhancing the operational efficiency and reducing energy consumption by minimizing the planning time and the path length. Future research will focus on the path planning of robotic arms in environments with dynamic obstacles.

## Figures and Tables

**Table 1:** Result comparison of different algorithms in Scenario I.

Algorithm	Average Path Length (mm)	Average Time (s)
RRT*	164.59	0.48
Informed-RRT*	162.58	0.56
RRT*-Connect	185.81	0.28
IRRT*-Connect	157.64	0.16

**Table 2:** Result comparison of different algorithms in Scenario II.

Algorithm	Average Path Length (mm)	Average Time (s)
RRT*	163.39	0.56
Informed-RRT*	162.28	0.76
RRT*-Connect	186.05	0.27
IRRT*-Connect	158.48	0.18

**Table 3:** Result comparison of different algorithms in Scenario III.

Algorithm	Average Path Length (mm)	Average Time (s)
RRT*	208.98	0.58
Informed-RRT*	206.13	0.68
RRT*-Connect	228.20	0.30
IRRT*-Connect	185.11	0.17

**Table 4:** Result comparison of different algorithms in Scenario IV.

Algorithm	Average Path Length (mm)	Average Time (s)
RRT*	210.28	1.12
Informed-RRT*	205.53	1.26
RRT*-Connect	214.81	0.28
IRRT*-Connect	181.78	0.18

**Table 5:** Robotic arm motion planning results.

Algorithm	Average Path Length (mm)	Average Time (s)	Planning Success Rate (%)
RRT*-Connect	366.58	8.49	86%
IRRT*-Connect	314.09	5.65	100%

## Data Availability

The data presented in this study are available on request from the corresponding author.
